# Exploring the evidence base for Communities of Practice in health research and translation: a scoping review

**DOI:** 10.1186/s12961-023-01000-x

**Published:** 2023-06-19

**Authors:** Janelle James-McAlpine, Sarah Larkins, Cate Nagle

**Affiliations:** 1 College of Medicine and Dentistry, James Cook University, 1 James Cook Drive, QLD 4811 Townsville, Australia; 2 College of Healthcare Sciences, James Cook University, 1 James Cook Drive, Townsville, QLD 4811 Australia; 3 Tropical Australian Academic Health Centre, James Cook University, 1 James Cook Drive, Townsville, QLD 4811 Australia

**Keywords:** Community of practice, Community of interest, Health research, Research translation, Knowledge transfer

## Abstract

**Background:**

The translation of research into healthcare practice relies on effective communication between disciplines, however strategies to address the gap between information sharing and knowledge transfer are still under exploration. Communities of Practice (CoP) are informal networks of stakeholders with shared knowledge or endeavour and present an opportunity to address this gap beyond disciplinary boundaries. However, the evidence-base supporting their development, implementation and efficacy in health is not well described. This review explores the evidence underpinning the use of CoP in health research and translation.

**Methods:**

A scoping review was undertaken using Arksey and O'Malley's methodological framework. A comprehensive search of health databases and grey literature was performed using keywords and controlled vocabulary. Studies were not restricted by date or research method.

**Results:**

A total of 1355 potentially relevant articles were identified through the global search strategy. Following screening, six articles were retained for analysis. Included studies were published between 2002 and 2013 in the United Kingdom (*n* = 3), Canada (*n* = 2) and Italy (*n* = 1). Three papers reported primary research; one used a quantitative methodology, one a qualitative, and one a descriptive evaluation approach. The three remaining papers explored seminal and evolving theories of CoP in the context of knowledge transfer and translation to the health sector.

**Conclusions:**

A paucity of evidence exists regarding the development and efficacy of CoP in health research and translation. Further empirical research is required to determine if communities of practice can enhance the translation of research into clinical practice.

**Supplementary Information:**

The online version contains supplementary material available at 10.1186/s12961-023-01000-x.

## Background

Communities of Practice (CoP) are defined as groups of individuals who come together to create an informal network of stakeholders with knowledge and expertise of a shared endeavour [1]. First described by Etienne Wenger in 1999 [2], CoP are increasingly recognised by industry as a valuable strategy for enhancing workplace interactions, innovation, and productivity. However, they are not a new concept. Throughout history, communities have evolved organically due to the human desire to gather together, draw on the knowledge of others and problem solve [3]. For example, artisans would come together to compare techniques and share discoveries, ultimately advancing the creative domain. Tradespeople would do the same in the functional sphere; religious leaders and philosophers gathered to debate the fine points of spirituality. Each made a significant contribution to their fields of expertise and the advancement of human knowledge [3].

By their nature, CoP were spontaneous and independent; however, the organisation of people into formal employment groups with specialised roles changed the way people interacted and learned [4]. The free-flowing exchange of ideas was curtailed due to time and intellectual property constraints, creating the concept of knowledge as a commodity [5]. As a result, the role of the masterful transformed from being the guardian of knowledge (ensuring knowledge transfer) to its keeper (protecting it from others), with a concomitant decline in broad community benefit.

The value of knowledge sharing has been increasingly acknowledged over the last three decades and has been recognised by industry leaders, including Hewlett Packard and the World Bank [1]. Introduction of CoP within these companies has enhanced their ability to address specific problems and distribute intellectual and social capital across their organisational networks. Such initiatives have improved productivity, promoted best practices, developed professional skills, attracted, and retained human resources [1].

Health is one industry that may benefit from the CoP approach. While natural networks form within disciplines such as nursing and medicine, these professions may be connected by referral rather than relational pathways. In particular, women’s health increasingly requires multidisciplinary coordination, with unique reproductive and socio-cultural implications adding an additional layer of collaborative complexity to provide appropriate, holistic, evidence-based care. An example of this is pregnancy, in which midwifery frequently intersects with dietetics, physiotherapy, social work, endocrinology, obstetrics, and neonatology. Each has a defined scope of practice, yet all may play a role in the reproductive continuum of women with diabetes. Despite this intersection, traditional interdisciplinary boundaries represent obstacles to the uptake of evidence-based practice and coordinated care [6]. Despite knowledge of the barriers to, and benefits of, the translation of research into practice, the health sector has proven to be a slow adopter of new strategies and its stakeholders resistant to change [7, 8]. Health services do not readily adopt practices without a thorough review of their evidence base, yet clinical practice frequently reflects habitual rather than evidence-based behaviours [9]. Therefore, a disconnection is evident between health services, clinical stakeholders, and researchers in complementary fields; strategies to address these barriers must be explored to optimise collaboration in multidisciplinary care.

In recognition of these needs, a nationally funded program in Australia is currently underway whose primary objective is to enhance research translation, and impact and reduce inequity in the field of women’s health, through creation of a national network of women working in women’s health and research. The Women’s Health Research Translation and Impact Network [10] seeks to enhance national and international networks, build health workforce capacity, and develop leaders in women’s health across nine priority women’s health areas—preconception, pregnancy, postpartum and intrapartum health, reproductive health, sexual health, mental health, chronic disease and preventative health, healthy lifestyle, violence and abuse, Indigenous health, and healthy ageing. Comprising seven National Health and Medical Research Council accredited Advanced Health Research and Translation Centres and three Centres for Innovation in Regional Health, the Network spans the continent, facilitating exploration of numerous strategies to enhance research translation and impact in women’s health.

One method employed locally was the creation and implementation of a CoP,: the impetus for this exploration of the literature. The aims of this scoping review were to explore the evidence underpinning communities of practice in women’s health research and translation, and to situate the findings in the broader context of the health sector. Systematic scoping reviews are commonly used to explore the range and nature of literature and evidence surrounding a subject of interest; the findings can assist in clarifying complex topics and refine future direction [11]. Given the diverse nature of CoP, their stakeholders, formats, and applications, a scoping review of the evidence base arising from their implementation and evaluation was warranted.

## Methods

### Literature search

The authors utilised Arksey and O'Malley’s theoretical framework to conduct this review in October 2021 [12]. A limited iterative search of MedLine and CINAHL complete was conducted in collaboration with an academic librarian to identify relevant peer-reviewed studies and to develop and refine the final search strategy. Preliminary search terms "community of interest", "community of practice", "women's health", "research translation", and "evidence-based practice" were combined with Medical Subject Headings (MeSH) terms using the Boolean operators "OR" and "AND".

A title and abstract review of retrieved articles and their indexing terms were performed; this was used to develop the final search terms for each database (Additional file 1: Appendix S1). A second comprehensive search was then undertaken across CINAHL complete, MedLine, Embase, Emcare, ProQuest, PsychInfo, PubMed, Scopus, and Google Scholar. Original primary empirical research (regardless of design) and pertinent grey literature including books, conference proceedings, working and white papers, research reports and theses, were considered eligible for inclusion; review papers were excluded, as were letters and commentaries. Reference and citation lists were searched for qualifying papers. No restrictions were applied to the date or language of publication.

This research was conducted according to the Preferred Reporting Items for Systematic Reviews and Meta-Analyses extension for Scoping Reviews (PRISMA-ScR) [13]. The review protocol has been registered with the Open Science Framework (10. 17605/OSF.IO/GNQ2H).

### Data extraction and analysis

Titles and abstracts were screened for relevance and data from eligible papers extracted by the first author. Full-text assessment of relevant papers was independently undertaken by two authors (JJ & CN). Reference lists and citations of the final inclusions were reviewed to ensure all literature informing their development was accounted for during the screening process.

Details relating to key article characteristics were extracted into a summary table, including authors, title, country of origin, approach, setting, population characteristics, objectives of the paper, and key findings related to the purpose of the review (Table 1). Papers reporting the results of primary research examining the efficacy of CoP on outcomes relating to network members were evaluated using indicators highlighted in each study (Table 1). This analysis was conducted separately to papers arising from review of the grey literature examining CoP theory. The latter were subjected to thematic and content analysis [14] using NVivo (v12) [15] to determine points of alignment.

**Table 1:** List and details of papers included in this review

Citation	Authors	Title	Country	Aim/objective of the paper	Approach	Setting	Population characteristics	Key findings
19	Barwick, M.A., Peters, J., & Boydell, K	Getting to uptake: Do communities of practice support the implementation of evidence-based practice?	Canada	To explore clinician practice, practice knowledge (of a new tool), and use of and satisfaction with implementation supports among clinicians participating in community of practice sessions versus clinicians engaging in usual practice Primary outcome—practice change as measured by clinician self-report and use of the CAFAS tool in practice	Randomised Control Trial Readiness for change, practice change, content knowledge, and satisfaction with and use of implementation supports were examined Likert scale assessments at baseline, midpoint and end-point Independent *t*-tests, two way repeated measures ANOVA	Clinicians from 6 consenting organizations were randomly assigned, clustered by organization, to either the CoP (*n* = 17 from 3 organizations) or PaU (*n* = 17 from 3 organizations) support conditions	Children’s mental health practitioners working in service provider organizations newly mandated to use the CAFAS outcome tool	No difference in readiness for change or reported practice change CoP participants demonstrated greater use of the tool in practice, better content knowledge and were more satisfied with implementation supports than PaU group 42% attrition rate Mostly female 66% of CoP 4 + sessions
20	Tagliaventi, M. R., & Mattarelli, E	The role of networks of practice, value sharing, and operational proximity in knowledge flows between professional groups	Italy	To formulate a grounded theory that is concerned with the factors that promote exchanges of knowledge between groups of professionals belonging to different networks of practice	Ethnographic study—grounded theory Observations mane for 21 weeks, five days a week, and 18 h per week on average. We observed each of the rooms three hours per week on average, for a total of 364 h	Radiation oncology unit of a major hospital in northern Italy	Doctors, radiotherapy technicians, medical physicists, and nurses	Both performing daily activities side-by-side and holding common values regarding the unit trigger the sharing of practices among professional groups that participate in different networks of practice
21	McDonald, P.W., & Viehbeck, S	From evidence-based practice making to practice-based evidence making: Creating communities of (research) and practice	Canada/ United States	Describes the CoP approach for enhancing exchange between researchers and practitioners Introduce the concept of Communities of Practice (CoPs) as a means of bridging the solitudes and overcoming limitations associated with current views of research translation	Three primary strategies (a) systematically recruiting researchers, program providers, and students interested in tobacco control; (b) creating a series of productivity tools; and (c) building social capital by creating smaller, focused CoPs (teams) on specific tobacco control practices or policy issues Came together on a common practice and then developed mutual goals and priorities through negotiation. Regular Web-based seminars, teleconferences, and occasional face-to-face meetings	North American Quitline Consortium	Researchers and program providers from across Canada and the United States who collectively focus on developing and sharing evidence to improve telephone-based counselling for smoking cessation	Annual symposium attendance up by 220% Rapid infusion of students and scientists Renewed commitment by experienced researchers to work more closely with one another Eight new multidisciplinary teams received seed grants to work on problems of mutual priority Dozens receiving and contributing to various communication vehicles The number of data sets being placed in the data repository is accelerating
22	Bate, S. P., & Robert, G	Knowledge management and communities of practice in the private sector: Lessons for modernising the National Health Service in England and Wales	United Kingdom	Examines how private sector knowledge management concepts and practices might help in the further development of public sector quality improvement initiatives	Examines NHS collaborative outcomes and an exploration of contemporary private sector practices with regard to knowledge management and communities of practice	NHS England and Wales	April 2000 to January 2002—the Cancer Services, Mental Health and Orthopaedic Services Collaboratives	Four areas for development suggested Information > knowledge Knowledge application > knowledge generation Explicit > tacit Contrived network > CoP
23	Kislov, R., Harvey, G., & Walshe, K	Collaborations for Leadership in Applied Health Research and Care: Lessons from the theory of communities of practice	United Kingdom	Discusses seminal theoretical literature on CoPs and previous empirical research on the role of these communities in healthcare collaboration	Combines the analytical and instrumental perspectives on communities of practice (CoPs) to reflect on potential challenges that may arise in the process of interprofessional and inter-organisational joint working	Collaborations for Leadership in Applied Health Research and Care	N/A	May be beneficial but further research needed
24	Thomson, L., Schneider, J., & Wright, N	Developing communities of practice to support the implementation of research into clinical practice	United Kingdom	To review the role of social networks in the translation of research into practice, propose a broader model of communities of practice (CoPs)	Presents an approach to supporting and developing CoPs around the specific context of an applied research programme in health and social care	Collaborations for Leadership in Applied Health Research and Care—Nottingham, Derbyshire and Lincolnshire, England involves practitioners, researchers, and service users		May be beneficial but further research needed

## Results

**Fig. 1:**
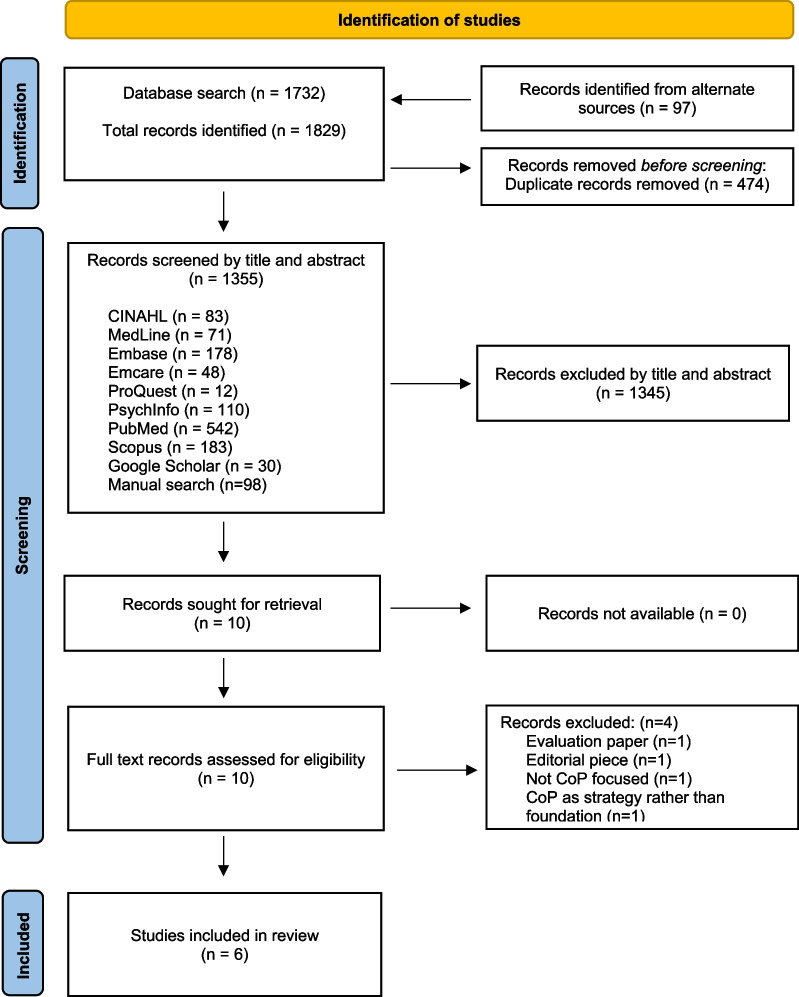
PRISMA ScR flowchart. Adapted from*:* Page MJ, McKenzie JE, Bossuyt PM, Boutron I, Hoffmann TC, Mulrow CD, et al. The PRISMA 2020 statement: an updated guideline for reporting systematic reviews. BMJ 2021;372:n71. doi: 10. 1136/bmj.n71. For more information, visit: http://www.prisma-statement.org/

A total of 1355 potentially relevant articles were identified through the global search strategy. Of these, none related specifically to women’s health; literature pertaining to the health field in general was sparse, therefore the scope of this review was expanded to include all eligible health related literature. Ten papers met the criteria for full-text review; six were retained for analysis (Fig. 1, Table 1). Of the four papers excluded, one was an evaluation paper [16], one an editorial piece [17], one not focused on CoP [18] and one utilised CoP as a strategy to address a specific intervention rather than examining the evidence behind the strategy [19] (Fig. 1, Additional file 2: Appendix S2).

Included papers were published in the United Kingdom (*n* = 3), Canada (*n* = 2) and Italy (*n* = 1) between 2002 and 2013. Three papers reported findings of primary research; of these, one used a randomised control trial design [20], one used an ethnographic approach culminating in a grounded theory [21] and one detailed a descriptive evaluation study [22]. The three remaining papers explored seminal and evolving theories of CoP in the context of knowledge transfer and translation within health services [23] and collaboratives [24, 25].

Barwick et al. (2009) characterised CoP as “a group of people who share knowledge, learn together, and create common practices” (p.17), with three pivotal elements shaping their structure and function—a domain of knowledge, a sense of community and shared practice. In their research, children's mental health clinicians from six participating organisations in Canada were randomised into one of two groups; the first received support for the implementation of a new Child and Adolescent Functional Assessment Scale (CAFAS) from a dedicated CoP, and the second received Practice as Usual (PaU) implementation support [20]. Sample sizes were even, with 17 participants from three organisations represented in each group. The majority of participants were female (89.2%) with an average of nine years of clinical experience. Knowledge and use of the CAFAS were measured, as were organisational readiness for change and satisfaction with implementation supports. Likert scale assessments were conducted at baseline, midpoint, and endpoints of the study; independent samples *t*-tests and repeated measures ANOVA were used to analyse data. No significant difference was found in readiness for change or changes in reported practice (*F*(1,17) = 11.7, *p* = 0.65). The CoP group demonstrated greater knowledge of the tool and its content (*t*(19) = 19.98, *p* = 0.01) and was more satisfied with implementation support than the PaU group (*t*(19) = 2.74, *p* < 001).

Tagliaventi and Mattarelli [21] conducted their ethnographic study in the radiation oncology unit of a major hospital in northern Italy. Central to their approach was the perspective that CoP share explicit (empirical and documented) and tacit (insights and intuition) knowledge through working alongside each other, a dynamic phrased as ‘proximity relations’ [21]. These authors also recognised three central tenets of these networks—reciprocity, joint enterprise, and shared repertoires. Aiming to formulate a grounded theory accounting for factors that promote knowledge exchange between disciplines in close proximity, the authors observed interactions between clinicians in a 'network of practice' five days a week for 18 weeks. From this, the researchers developed proximity and knowledge-related matrices, demonstrating the relationship between sharing objects, spaces, activities and transdisciplinary knowledge transfer.

A total of 364 h of observed knowledge exchange resulted in 11,396 recorded interactions between doctors, radiotherapy technicians, and medical physicists. Their analysis highlighted the importance of shared space, resources, and goals for knowledge transfer between professional groups; they propose that this diffusion of knowledge at interdisciplinary boundaries creates a new type of "organisational citizenship behaviour"—the constructive actions and behaviours that contribute to a beneficial workplace culture [21]. Tagliaventi and Mattarelli [21] also proposed that those who initiate knowledge transfer under these conditions prompt reciprocity from those with whom they engage; these behaviours then infiltrate the organisation, facilitating sustainable practice change.

McDonald and Viehbeck [22] described CoP frameworks as “a group of people who share a common interest in a particular practice or problem” (p.142), whose activities not only share best practice, but create knowledge to advance practice. Using this foundation, the authors integrated CoP in the North American Quitline Consortium. This organisation took a proactive approach to introducing the CoP; evaluation was also conducted to determine the ability of the CoP to overcome barriers to research translation, such as isolation and communication. To this end, researchers, providers, and students were systematically recruited to the CoP; members developed productivity tools, built social capital, established mutual goals and priorities, and interacted through regular webinars, teleconferences, and face-to-face meetings. Evaluation of the model found enhanced engagement with professional development, rapid infusion of students and scientists to the consortium, renewed commitment to collaboration, enhanced communication, an increase in available datasets and successful funding applications for projects of mutual priority [22].

The remaining three papers described the application of the concepts underpinning CoP in the context of health. Kislov, Harvey and Walsh [24] define a CoP as “a group of people who share a concern, a set of problems, or a passion about a particular topic, and who deepen their understanding and knowledge of this area by interacting on an ongoing basis” (p.2); Thomson, Schneider and Wright [25] use the more succinct definition “groups of people informally bound together by shared expertise and passion for joint enterprise”—as their conceptual model. These two papers described Collaborations for Leadership in Applied Health Research and Care (CLAHRCs); a CoP variation modelled on aligned principles. An English initiative commencing in 2008, nine CLAHRCs were created across multiple jurisdictions to narrow the gap between research and practice through enhanced collaboration between health services and educational institutions [24]. The first of these papers describes the support and development requirements of CoP in an applied health and social care research program in the Nottingham, Derbyshire, and Lincolnshire CLAHRC [25]; the other discusses the evidence surrounding the role of CoP in the generalised CLAHRC model [24].

The sixth paper explored knowledge management (KM) concepts and practices used by private enterprise and their potential contribution to National Health Service (NHS, United Kingdom) quality improvement initiatives. Citing evidence arising from the application of 'collaboratives' in mental health, cancer and orthopaedic services, Bate and Robert [23] considered a CoP to be “where people share their experiences and knowledge in free-flowing creative ways so as to foster new approaches to problem solving and improvement, help drive strategy, transfer best practice, develop professional skills and help companies recruit and retain staff” (p. 652). The authors proposed four possible areas for knowledge management development: the translation of information to knowledge; from knowledge application to knowledge generation; the transformation of tacit to explicit knowledge; and the journey from contrived networks to communities of practice [23].

Thematic analysis highlighted five threads central to these papers: (i) CoP characteristics and capabilities; (ii) CoP infrastructure requirements; (iii) knowledge transfer and translation; (iv) barriers to the creation and function of CoP in practice; and (v) the strength of the evidence base underpinning their use (Table 2).

**Table 2:** Thematic analysis of non-empirical papers

Theme	Kislov et al. [24]	Thomson et al. [25]	Bate and Robert [23]
Characteristics and capabilities	• (The CoP approach) "demonstrated to enhance interprofessional clinical practice, facilitate quality improvement, encourage buy-in among participants, promote knowledge transfer" • Require a domain of knowledge, defining a set of issues, community of people who care about this domain, create, expand and exchange knowledge, develop individuals, self-selection, passion, commitment, and identification with the group and its expertise, evolve and end organically	• “Groups of people informally bound together by shared expertise and passion for joint enterprise” • Include social interaction, knowledge-creation, knowledge-sharing, and identity-building • Builds on the powerful influences of natural networks—groups of clinicians who interact professionally to share information, support, consult, refer and jointly manage patients	• Relies upon the adaptation of existing knowledge to multiple settings to accomplish common goals • Enable a wide range of professionals in a large number of organisations to come together to learn and ‘harvest’ good practice from each other, • Create horizontal networks cutting across hierarchical and relatively isolated organisations • Empower relatively junior staff to take ownership for solving local problems by working with clinicians who have taken change leadership roles
Infrastructure	• Requires knowledge brokers, boundary objects, boundary interactions among people from different CoPs • Boundaries are fuzzy • Link those who conduct applied health research with all those who use it	• Open to change and change management • Bottom-up strategy • Map participants and identify missing stakeholders • Development informal and spontaneous, • Participants possessing qualities and characteristics necessary to develop and sustain community • Financial support for providing facilitators, materials, paying for backfill for clinicians’ absences when they attended CoPs • Creation of dedicated posts to support evidence-based practice training within healthcare organisations • A continuous learning culture	
Knowledge transfer and translation	• A transdisciplinary project can act as a bridge to enhance knowledge transfer and learning at the boundaries • Shaped strongly by the personal, political, and professional agendas of the participants	• Approach to knowledge translation based on socially situated learning • Sharing evidence-based examples more likely to change practice than disseminating new knowledge • Guiding existing practices within natural networks more effective than directing clinicians to change their practice	• Knowledge dissemination and transferability only occur when there is a collective identity
Challenges	• Incompatible epistemic cultures • Biomedical paradigms vs ethnographic approaches • Formalisation of organic CoPs can disrupt knowledge-sharing • Boundaries between clinical and management practice		• Implementation gap • Maintaining motivation and commitment in the face of clinical demands • Identifying skilled frontline staff to lead and participate • May not lead to sustained change—'single-loop learning'
Evidence-base	• Development, functioning, and effects of multi-professional and multi-agency CoPs remains under-researched	• Need to assess how closely the theory of developing these CoPs matches reality • Ongoing monitoring and evaluation of how CoPs develop around the clinical themes important	• Use of CoPs driven more by faith than research • Scarcity of empirical work on the 'people issues'

### CoP characteristics and capabilities

CoP evolve spontaneously and end organically [24]; they provide a means to create horizontal networks, minimising the effect of hierarchy on knowledge transfer [23]. This supports the development and empowerment of junior stakeholders, linking them with those with leadership experience [21]. CoP create opportunities for adapting and adopting knowledge and expertise between complementary disciplines [21, 24], enhancing clinical practice, facilitating continuous quality improvement and accomplish common goals [24]. Community members are characterised by self-selection, passion for the issues central to the community, commitment to and identification with the community as a whole [24], and are supported by social nature of natural networks [25].

### CoP infrastructure requirements

Infrastructure requirements include fluid boundaries, informal and spontaneous community development, bridges between research and practice supported by strategic facilitators, shared space and resources, proximity relations [24], network mapping, identification of gaps, a bottom-up approach, desire for change and a continuous learning culture [25]. Network facilitators benefit from financial support [25].

### Knowledge transfer and translation

CoP are strongly shaped by the personal, professional and political agendas of their members [24]. However, knowledge transfer only occurs where a collective identity exists [21]. In turn, the collective identity is informed by alignment of individual values [21], and knowledge transfer occurs within the network—a socially-situated learning paradigm [25]. This alignment acts as a bridge between disciplines, with knowledge sharing occurring at transdisciplinary boundaries [24].

### Barriers to the creation and function of CoP in practice

Identification of skilled key facilitators is essential to the function of CoP [21]. However, maintenance of and commitment to a CoP for skilled personnel may be difficult in the face of staff shortages, and disparities between CoP, research, and health service priorities [21]. These disparities extend to differences in the creation of knowledge between science and clinical practice, biomedical and ethnographic approaches, hierarchical and horizontal organisation, and reactive versus proactive practice [21, 24].

### The strength of the evidence base underpinning their use

Little empirical research has been conducted specifically examining the efficacy of CoP or the factors that contribute to their success [21]. This includes research into internal influences such as the personal, professional, or political agendas of individual network members, or their ability to affect change within and external to the CoP. Further, factors external to the CoP, yet intrinsic to the health sector, such as funding, capacity, culture, resources and sustainability are not well understood [24, 25]. As such, CoP in the health sector are supported by belief rather than evidence [21].

The five most common words found in the content analysis were: knowledge (*n* = 56); practice (*n* = 49); change (*n* = 30); organisations (*n* = 27); and development (*n* = 23; Table 3).

**Table 3:** Content analysis—most common words found in non-empirical papers

Word	Count	Similar words
Knowledge	56	Initial, know, knowledge, learn, learning
Practice	49	Applied, apply, commitment, expert, practical, practice, practices, skilled, skills, using, virtual
Change	30	Change, changes, changing, convert, deepen, exchange, transfer, transferability, transferred, variety
Organisations	27	Coordination, directing, established, establishing, formation, organic, organically, organisation, organisational, organisations, systems
Development	23	Arising, develop, developing, development, education, educational, evolve, evolving, produce, producing, training

## Discussion

Communities of practice are groups of people of shared expertise and passion who, through informal or intentional social interaction, build common identity, create, exchange and expand knowledge, develop research and practice capacity, capability and confidence, and affect change through improving their collective wisdom in relation to a joint enterprise [2, 20–25]. Variations of this definition are cited by all included articles and supported by keyword frequency analysis of non-empirical papers. However, while there is little disagreement between authors regarding the essence of communities of practice, theoretical and conceptual frameworks have not significantly developed beyond those proposed in Wenger's seminal literature.

Wenger's theory of socially situated learning proposes that knowledge transfer is essentially a social process, and that this pedagogy is situated within specific social and physical environments, such as CoP [2]. This theory has withstood scrutiny from a broad cross-section of business and industry, however, is yet to be fully appreciated by the health sector. While the natural networks that develop in health settings exhibit points of similarity with CoP, their potential remains largely untapped; therefore, their benefit in knowledge transfer and organisational culture is yet to be realised. Furthermore, little primary research has been conducted that examines the degree of learning or engagement, personal or professional development in the health sector.

Only three empirical studies were found to qualify for inclusion in this review; each of these adopted different methodologies, and each was conducted and published prior to the introduction of stringent implementation evaluation methods, such as the Consolidated Framework for Implementation Research [26]. As such, it is not possible to draw conclusions regarding best practice or evidence-based approaches to CoP creation and implementation in healthcare. Further, the process of formalising CoP infrastructure into a repeatable framework negates the informality and authenticity that appear to be essential for their success.

The informal nature of CoP may be problematic in terms of defining outcomes measures, which may be fluid and dependent on the purpose and scope of the CoP in question. For example, while CoP members in Barwick et al.’s [20] RCT demonstrated a better understanding of the CAFAT and reported higher satisfaction with implementation support than the control group, findings were limited by the small sample size (*n* = 20) and inequity of CoP representation across study sites. The descriptive evaluation by McDonald and Viehbeck [22] reported enhanced engagement, research input and research outputs anecdotally rather than empirically, further highlighting the challenges associated with consistent measurement [22]. While their introduction of CoP was strategic and methodical, the study's value and the transferability of its findings to practice are limited.

Further, elucidating the human factors driving CoP engagement is challenging given that, by definition, the membership is not subject to oversight or censure, and that participants move in and out of the group liaising with different group members according to their interests or needs. Personal, professional and organisational politics each influence an individual’s willingness to engage, as demonstrated in the CoP arm of the RCT; these participants had their travel costs remunerated and backfill was provided for their clinical positions. Despite this financial support, a 42% attrition rate was recorded for the project. While the authors did not propose theories for this lack of retention it appears that disengagement may not be financially motivated. These findings demonstrate some of the challenges to the implementation and consistency of CoP in health services; they also highlight the need for flexibility and fluidity, and an understanding of the needs and motivations of both the health service and community members. Given that people are central to such communities, more work needs to be undertaken to understand the 'people issues' that influence the effectiveness of CoP [23]. Individual motivations, politics, skill sets, biases, and professional agendas are central to the collective identity [23, 24]; therefore, their influence needs to be understood on a local level in order to determine their generalisability.

Tagliaventi and Mattarelli's [20] grounded theory provided valuable insights into the role of personal proximity and boundary objects in transdisciplinary communication and knowledge transfer. This method exhibited limitations, however, as the research space was physically situated within one department in a single hospital. Only explicit knowledge transfer was examined in this study, given that tacit knowledge transfer is difficult when not sharing a physical space. These limitations suggest that the application of this grounded theory may be of limited value to more broad and dispersed networks. However, this theory applied in a specific multidisciplinary environment may assist to enhance transdisciplinary relations and address barriers to knowledge transfer across traditional boundaries. Further, these limitations may be addressed by hybrid or online approaches to CoP creation. Digital communities have proven beneficial in occupations or environments that demonstrate gender inequity similar to those experienced by women in the health sector [27]. Therefore, capitalising on the gender differences inherent to socially situated learning may present an opportunity to enhance capacity and confidence in women working in women’s health, research, and translation; this cooperative approach may result in enhanced knowledge transfer and improved women’s health outcomes [28].

Despite the variety of research methods utilised in the included studies, they all agreed that the evidence underpinning the use of CoP in health research and practice is limited; no evidence of their implementation and efficacy in women’s health specifically was found. However, the methodological disparity highlights the range of research methods applicable to CoP evaluation in terms of efficacy and transdisciplinary knowledge transfer. Additionally, factors external to CoP dynamics may influence the feasibility and sustainability of CoP within health services, and therefore their efficacy in relation to knowledge acquisition and transfer. The strengths and challenges of transdisciplinary and multi-agency CoP in the health sector remain under-researched and are deserving of more vigorous evaluation [24] Therefore, both qualitative and quantitative methods must be used to measure outcomes, learning, engagement, and the factors that influence such pivotal outcome measures. Work of this design will enable evaluation of how closely the theory and practice of CoP development align [25]. Conduct of CoP evaluation studies within pre-defined and established implementation science evaluation frameworks may enhance the evidence base supporting their role in knowledge transfer and the translation of research into practice [26].

## Conclusion

A paucity of evidence exists regarding the development and efficacy of communities of practice in health research and translation. Further empirical research is required to determine the optimal structures and supports required to bridge the gap between information sharing and knowledge transfer in the translation of research into clinical practice.

## Supplementary Information

**Additional file 1: Appendix S1.** Search terms by database.

**Additional file 2: Appendix S2.** List of papers excluded from this review.

## Data Availability

All data pertaining to this review have been included as appendices.

## Abbreviations

CoP: Community of practice
PAU: Practice as usual
