# Supplementary material for: Exploring the evidence base for Communities of Practice in health research and translation: a scoping review
Source: Health Res Policy Syst. 2023 Jun 19;21:55. doi: 10.1186/s12961-023-01000-x (PMC10278351; doi:10.1186/s12961-023-01000-x)
Supplement: Supplementary file 1 — Additional file 1: Appendix S1. Search terms by database. [file 12961_2023_1000_MOESM1_ESM.docx]

Appendix S1: Search terms by database

| Database | Search term 1 AND | Search term 2 AND | Search term 3 AND/OR | Search term 4 |
| --- | --- | --- | --- | --- |
| Medline | "communities of interest" or "communities of practice" or "community of interest" or "community of practice" or "collaborative networks" or "collaborative network" or networks of excellence or "Centres of Excellence" or "Knowledge Networks" or "Networks of Expertise" or "Special Interest Groups" or network of excellence or "Centre of Excellence" or "Centers of Excellence" or "Centers of Excellence" or "Knowledge Network" or "Network of Expertise" or "Special Interest Group" or CoPs or COIs or CoP's or COI's | "woman's health" or "women's health" or "womens health" or "maternal health" or health | research or "research translation" | "evidence-based practice" or "evidence based practice" |
| CINAHL complete | "communities of interest" OR  "communities of practice" OR  "community of interest" OR  "community of practice" OR  "collaborative networks" OR  "collaborative network" OR networks  of excellence OR "Centres of  Excellence" OR "Knowledge  Networks" OR "Networks of  Expertise" OR "Special Interest  Groups" OR network of excellence  OR "Centre of Excellence" OR  "Centers of Excellence" OR "Centers  of Excellence" OR "Knowledge  Network" OR "Network of Expertise"  OR "Special Interest Group" | Women's Health" OR  "Maternal-Child Health" OR Health | Research+ | "evidence-based practice" or "evidence based practice" |
| Embase, Emcare, Proquest, PsychInfo, PubMed | "communities of interest" or "communities of practice" or "community of interest" or "community of practice" or "collaborative networks" or "collaborative network" or networks of excellence or "Centres of Excellence" or "Knowledge Networks" or "Networks of Expertise" or "Special Interest Groups" or network of excellence or "Centre of Excellence" or "Centers of Excellence" or "Centers of Excellence" or "Knowledge Network" or "Network of Expertise" or "Special Interest Group" or CoPs or COIs or CoP's or COI's | "woman's health" or "women's health" or "womens health" or "maternal health" or health | research or "research translation" | "evidence-based practice" or "evidence based practice" |
| Scopus | "communit* of interest" OR "communit* of practice" "collaborative network*" OR "network* of excellence" OR "centre* of excellence*" OR "special interest group*" "Knowledge Network*" OR "Network* of Expertise" | "woman* health" OR "women* health" OR health | research OR "research translation" | "evidence-based practice" OR "evidence based practice" |
| Google Scholar | 'communit* of interest' OR communit* of practice | 'wom* health' OR health | 'wom* health research' OR ‘health research’ | research translation |
