# Supplementary material for: Exploring the evidence base for Communities of Practice in health research and translation: a scoping review
Source: Health Res Policy Syst. 2023 Jun 19;21:55. doi: 10.1186/s12961-023-01000-x (PMC10278351; doi:10.1186/s12961-023-01000-x)
Supplement: Supplementary file 2 — Additional file 2: Appendix S2. List of papers excluded from this review. [file 12961_2023_1000_MOESM2_ESM.docx]

Appendix S2: List of papers excluded from this review

| Authors | Year | Title | Journal | Reason for exclusion |
| --- | --- | --- | --- | --- |
| Anderson-Carpenter, K. D., Watson-Thompson, J., Jones, M., & Chaney, L. | 2014 | Using communities of practice to support implementation of evidence-based prevention strategies. | Journal of Community Practice | Reported on outcomes of community practice interventions rather than the efficacy of CoPs. Not centred on research translation |
| Bowen, S., Botting, I., Graham, I. D., MacLeod, M., Moissac, D., Harlos, K., Leduc, B., Ulrich, C., & Knox, J. | 2019 | Experience of health leadership in partnering with university-based researchers in Canada - a call to "re-imagine" research. | International Journal of Health Policy Management | Examined relationships between senior health personnel and health system research. Does not contribute to CoP evidence |
| Nagykaldi, Z. | 2014 | Practice-based research networks at the crossroads of research translation | Journal of the American Board of Family Medicine | Editorial |
| Ranmuthugala, G., Cunningham, F. C., Plumb, J. J., Long, J., Georgiou, A., Westbrook, J. I., & Braithwaite, J. | 2011 | A realist evaluation of the role of communities of practice in changing healthcare practice. | Implementation Science | Study protocol |
